# Insights into student assessment outcomes in rural clinical campuses

**DOI:** 10.1186/s12909-019-1828-z

**Published:** 2019-10-18

**Authors:** Boaz Shulruf, Gary Velan, Lesley Forster, Anthony O’Sullivan, Peter Harris, Silas Taylor

**Affiliations:** Office of Medical Education, University of New South Wales, Sydney, 2052 Australia

**Keywords:** Assessment, Rural medical education, OSCE, Australia

## Abstract

**Background:**

There is an ongoing debate about the impact of studying medicine in rural vs. metropolitan campuses on student assessment outcomes. The UNSW Medicine Rural Clinical School has five main campuses; Albury-Wodonga, Coffs Harbour, Griffith, Port Macquarie and Wagga Wagga. Historical data of student assessment outcomes at these campuses raised concerns regarding potential biases in assessment undertaken, as well as the availability and quality of learning resources. The current study aims to identify the extent to which the location of examination (rural versus metropolitan) has an impact on student marks in OSCEs.

**Methods:**

Assessment data was employed for this study from 275 medical students who sat their final examinations in Years 3 and 6 of the undergraduate Medicine program at UNSW in 2018. The data consists of matched student assessment results from the Year 3 (Y3) MCQ examination and OSCE, and from the Year 6 (Y6) MCQ, OSCE and management viva examinations. The analysis used Univariate Analysis of Variance and linear regression models to identify the impact of site of learning and site of examination on assessment outcomes.

**Results:**

The results demonstrate that neither site of learning nor site of examination had any significant impact on OSCE or Management Viva assessment outcomes while potential confounders are controlled.

**Conclusion:**

It is suggested that some of the supposed disadvantages inherent at rural campuses are effectively mitigated by perceived advantages; more intensive interaction with patients, the general and medical communities at those sites, as well as effective e-learning resources and moderation of assessment grades.

## Background

The need to enhance and sustain the medical workforce in rural communities has been one of the most important health objectives of many countries, including Australia, New Zealand and Canada. Traditionally, medical schools have addressed rural communities’ need for more doctors by implementing student selection processes that aimed to identify those who were likely to pursue a medical career in rural regions [1]. Despite these efforts, the evidence regarding the effectiveness of the selection process alone to boost the number of doctors settling in rural communities is not promising [1, 2].

An alternative or complementary method for attracting medical students to pursue their career in rural communities has been deliberate exposure to rural medicine during medical studies [3–5]. A recent study suggests that even a short clinical learning experience in a rural community has a positive effect on medical students from metropolitan communities and increases the likelihood they would choose in future to practise medicine in rural regions [6]. It has also been suggested that the longer the students stay rural the more likely they are to practice rural [7].

Many medical schools in Australia, New Zealand, Canada and elsewhere have developed rural clinical schools where students live and study in these rural communities for a significant length of time during their medical training (for example: [1, 8–10]).Consequently, enhancing medical students’ experience in rural placements has become an important objective for these schools. With this in mind, there is evidence suggesting that the clinical learning experience of students in rural settings also has positive impacts on their performance in clinical skills assessments [11]. It has also been reported that students’ learning experience in rural settings is associated with subsequent rural career choices [11, 12]. Possible explanations for such impact of rural clinical experiences might be related to the nature of rural settings, whereby students are most commonly engaged in more intimate clinical settings learning in smaller groups which foster greater personal interactions with clinicians and the community, and experiencing a lower ratio of medical students to available patients [13–15].

Despite the evidence supporting the efficacy of rural clinical learning experiences on outcomes for medical students, it remains open to conjecture whether the grades of students who study and are assessed in rural clinical settings are related more to the learning experience, or whether the clinical skills assessment undertaken itself plays a role [16]. For example, compared with metropolitan settings, in rural communities student engagement with clinical and academic staff is more intensive, since they live and work within the same community and the daily interaction either within the working/learning environment or after hours is often unavoidable [17, 18].On the other hand, studies on potential biases impacting on examiner marking in OSCEs suggest that such examiner familiarity with students has a positive bias on student grades [19, 20].

The current study aims to identify the extent to which the location of examination (rural as compared with metropolitan) has an impact on student marks in OSCEs.

## Methods

### Setting

The UNSW Medicine Rural Clinical School has five main campuses – at Albury-Wodonga, Coffs Harbour, Griffith, Port Macquarie and Wagga Wagga. With the exception of Port Macquarie where students can undertake their entire course, students can spend up to 4 years of the 6-year Medicine program at a rural campus where they are taught predominantly by local clinicians and a smaller group of clinical academics. In total, approximately 50 students complete Year 3 in a rural campus and around 70 students undertake the last 2 years of their studies and sit their final exams in these country settings. UNSW Medicine has four main metropolitan campuses all based in Sydney.

This study employed assessment data from one cohort of medical students (*N* = 275) who sat their final examinations in Year 3 (in 2015) and Year 6 (in 2018) of the undergraduate Medicine program at UNSW. The data was received from the student administration office in February 2019. Data consist of matched student assessment results (total examination marks) from the Year 3 (Y3) MCQ examination and OSCE, as well as from the Year 6 (Y6) MCQ, OSCE and management viva examinations. The MCQ examinations were all conducted and answered online, and all students participated in the MCQ examination at the identical time independent of their rural or metropolitan site. For the OSCE and management viva examinations, questions are selected from an Assessment Item Bank so that all questions are the same at all rural and metropolitan sites for examinations held at the same date and time. Examination supervisor and examiners guidelines are prepared and distributed to all examination sites in advance. The rural OSCE and management viva examinations are always held at the same date and time as metropolitan sites. Most examiners remain in their teaching / clinical settings, although a few examiners move sites. For OSCE and viva examinations, the grading is performed on iPads and the grading criteria are identical for all metropolitan and rural sites. Following completion of the examinations the iPads are synchronised and all grading data and comments are electronically downloaded to the UNSW Medicine administration site in Sydney. In addition, data include site of clinical learning (home site) in Y3 and Y6, as well as the site for each examination, including whether the site was rural or metropolitan. Due to ethical requirements, the data does not include any individual characteristics of either students or examiners. Note that student and examiner individual characteristic data was not necessary for the analysis.

### Statistical analysis

The analysis employed Univariate Analysis of Variance to identify the impact of site of learning and site of examination on assessment outcomes. Partial η 2[21] was used to compare the relative effect size (impact) of the independent variable on each assessment outcome (dependent variable). It is noted that partial η^2^ is not the most accurate measure of effect size compared with Cohen’s d when more than one category is assessed. However, as a *relative* measure partial η^2^ is practical and acceptable [22]. The large number of sites reduces the statistical power and therefore additional analysis took place where type of site (Rural/ Metropolitan) was used rather than the named site. This was undertaken under the assumption that rural campuses share some common features which make them different to Metropolitan campuses [4, 6, 17]. This also required adding a variable indicating whether the students were assessed in the same or different site where they had studies. Using features (rural/Metropolitan and same/different) increased the statistical power as well as made this analysis more generalisable.

Multiple linear regressions were used to identify the impact of sites of learning and examination (rural/metropolitan) on assessment outcomes.

It is acknowledged that education data may not always fully meet the assumption of normality. Yet, in reality if the data distribution is not extreme the risk of Type 1 & 2 errors is negligible [23]. The MCQ, OSCE and Management Viva data were tested for normality and the OSCE and Management viva appeared to not meeting the normal distribution but the breach was minimal (Kurtosis <│4.65│; and Skewness <│1.53│). That deviation from the normal distribution is small and should have not impacted the adequacy of the analysis [23]. The independent variables were tested for colinearity and found not to collinear (VIF < 2.4). The analysis was undertaken using SPSS v24 [24].

## Results

The impact of clinical assessment site on OSCE results was the largest among the variables considered, yet not statistically significant (partial η^2^ = .745; *p* = .08) once impacts of home site and MCQ results are accounted for. Home site did not have any significant impact on OSCE results. However, MCQ results had small, yet statistically significant (partial η2 = .157; *p* < .001) impact on the OSCE results once home and examination sites are controlled for (Table 1). It was also found that home site in either Y3 or Y6 had no significant impact on Y6 MCQ’s. The only impact on Y6 MCQ results was of the Y3 MCQ results (partial η^2^ = .223, *p* < .001) (Table 2).

**Table 1:** Factors impacting Y3 OSCE results: univariate analysis, tests of between-subjects effects

Source	Type III Sum of Squares	df	Mean Square	F	Sig.	Partial η2
Intercept	186.036	1	186.036	23.843	0.000	0.094
	1783.433	228.566	7.803^a^			
Y3 MCQ	331.269	1	331.269	42.337	0.000	0.157
	1784.013	228	7.825^b^			
Y3 OSCE Site	66.408	4	16.602	3.877	0.080	0.746
	22.568	5.27	4.283^c^			
Y3 Home Site	6.229	4	1.557	0.353	0.834	0.189
	26.654	6.039	4.414^d^			
Y3 OSCE Site * Y3 Home Site	26.447	6	4.408	0.563	0.759	0.015
	1784.013	228	7.825^b^			

^a^.003 MS(Y3 Home Site) + .001 MS(Y3 OSCE Site * Y3 Home Site) + .996 MS(Error)

^b^MS(Error)

^c^1.037 MS(Y3 OSCE Site * Y3 Home Site) - .037 MS(Error)

^d^.998 MS(Y3 OSCE Site * Y3 Home Site) + .002 MS(Error)

**Table 2:** Factors impacting Y6 MCQ results: univariate analysis, tests of between-subjects effects

Source	Type III Sum of Squares	df	Mean Square	F	Sig.	Partial η2
Intercept	144.988	1	144.988	4.478	0.035	0.02
	7262.994	224.297	32.381^a^			
Y3 MCQ	2034.236	1	2034.236	63.036	0.000	0.223
	7099.576	220	32.271^b^			
Y6 Home Site	140.892	3	46.964	1.459	0.236	0.074
	1766.526	54.872	32.194^c^			
Y3 Home Site	409.823	9	45.536	1.416	0.236	0.346
	775.594	24.123	32.151^d^			
Y6 Home Site * Y3 Home Site	481.783	15	32.119	0.995	0.461	0.064
	7099.576	220	32.271^b^			

^a^.008 MS(Y3 Home Site) - .000 MS(Y6 Home Site * Y3 Home Site) + .992 MS(Error)

^b^MS(Error)

^c^.508 MS(Y6 Home Site * Y3 Home Site) + .492 MS(Error)

^d^.787 MS(Y6 Home Site * Y3 Home Site) + .213 MS(Error)

The next three analyses aimed to identify factors impacting each of the three main assessments undertaken at the end of Y6. These are high-stakes examinations, since passing all three of them is required for graduation.

The impact of examination site in Y6was estimated using Univariate analysis as follows: Dependent variable: Score in the type of examination (OSCE, MCQ, Viva); Fixed Factor: Site of the examination for each type of examination (as above); Random Factors: Y3 home site and Y6 home site; and Covariate: Y3MCQ results, Y3OSCE results, and two of the three Y6 results that are not the dependent variable, i.e. two ofY6MCQ, OSCE and Management Viva. In this way, the impact of the examination site on the examination results was assessed while most of the other important variables are controlled.

The results demonstrate that once all other variables are held constant (i.e. controlled) the examination site had no significant impact on the examination results (Tables 3, 4 and 5).

**Table 3:** Factors impacting Y6 OSCE results: univariate analysis, tests of between-subjects effects

Source	Type III Sum of Squares	df	Mean Square	F	Sig.	Partial η^2^
Intercept	117.048	1	117.048	5.177	0.024	0.029
	3894.967	172.275	22.609^a^			
Y3 MCQ	0.175	1	0.175	0.008	0.930	0
	3880.973	171	22.696^b^			
Y3 OSCE	404.214	1	404.214	17.81	0.000	0.094
	3880.973	171	22.696^b^			
Y6 MCQ	148.939	1	148.939	6.562	0.011	0.037
	3880.973	171	22.696^b^			
Y6 Management Viva	1087.256	1	1087.256	47.906	0.000	0.219
	3880.973	171	22.696^b^			
Y6 OSCE Site	324.523	6	54.087	75.24	0.991	1
	0.001	0.002	.719^c^			
Y3 Home Site	134.219	9	14.913	3.674	0.500	0.982
	2.473	0.609	4.059^d^			
Y6 Home Site	5.375	1	.	.	.	.
	.	.^e^	.			
Y6 OSCE Site * Y3 Home Site	523.901	25	20.956	0.434	0.962	0.479
	569.68	11.799	48.284^f^			
Y6 OSCE Site * Y6 Home Site	98.392	4	24.598	0.534	0.713	0.142
	593.056	12.878	46.051^g^			
Y3 Home Site * Y6 Home Site	10.55	4	2.637	0.054	0.994	0.018
	563.623	11.507	48.980^h^			
Y6 OSCE Site * Y3 Home Site * Y6 Home Site	553.401	11	50.309	2.217	0.016	0.125
	3880.973	171	22.696^b^			

^a^.006 MS(Y3 Home Site) + .005 MS(Y6 Home Site) + .000 MS(Y6 OSCE Site * Y3 Home Site) + .002 MS(Y6 OSCE Site * Y6 Home Site) - .004 MS(Y3 Home Site * Y6 Home Site) - .002 MS(Y6 OSCE Site * Y3 Home Site * Y6 Home Site) + .992 MS(Error)

^b^MS(Error)

^c^.768 MS(Y6 OSCE Site * Y3 Home Site) + .982 MS(Y6 OSCE Site * Y6 Home Site) - .815 MS(Y6 OSCE Site * Y3 Home Site * Y6 Home Site) + .066 MS(Error)

^d^.594 MS(Y6 OSCE Site * Y3 Home Site) + .469 MS(Y3 Home Site * Y6 Home Site) - .297 MS(Y6 OSCE Site * Y3 Home Site * Y6 Home Site) + .234 MS(Error)

^e^Cannot compute the error degrees of freedom using Satterthwaite’s method

^f^.927 MS(Y6 OSCE Site * Y3 Home Site * Y6 Home Site) + .073 MS(Error)

^g^.846 MS(Y6 OSCE Site * Y3 Home Site * Y6 Home Site) + .154 MS(Error)

^h^.952 MS(Y6 OSCE Site * Y3 Home Site * Y6 Home Site) + .048 MS(Error)

**Table 4:** Factors impacting Y6 Management Viva results: univariate analysis, tests of between-subjects effects

Source	Type III Sum of Squares	df	Mean Square	F	Sig.	Partial Eta Squared
Intercept	2.599	1	2.599	0.061	0.806	0
	7514.343	175.596	42.793^a^			
Y3 MCQ	43.184	1	43.184	1.003	0.318	0.006
	7530.942	175	43.034^b^			
Y3 OSCE	5.427	1	5.427	0.126	0.723	0.001
	7530.942	175	43.034^b^			
Y6 MCQ	456.106	1	456.106	10.599	0.001	0.057
	7530.942	175	43.034^b^			
Y6 OSCE	1834.285	1	1834.285	42.624	0	0.196
	7530.942	175	43.034^b^			
Y6 Management Viva Site	190.866	6	31.811	1.171	0.414	0.498
	192.759	7.094	27.172^c^			
Y3 Home Site	80.564	9	8.952	0.264	0.979	0.081
	915.046	27.021	33.864^d^			
Y6 Home Site	23.224	1	23.224	1.754	0.377	0.584
	16.528	1.248	13.242^e^			
Y6 Management Viva Site * Y3 Home Site	837.784	25	33.511	1.425	0.237	0.698
	362.808	15.425	23.521^f^			
Y6 Management Viva Site * Y6 Home Site	51.644	4	12.911	0.593	0.675	0.174
	244.562	11.228	21.782^g^			
Y3 Home Site * Y6 Home Site	70.709	4	17.677	0.888	0.513	0.309
	157.758	7.928	19.899^h^			
Y6 Management Viva Site * Y3 Home Site * Y6 Home Site	134.634	7	19.233	0.447	0.871	0.018
	7530.942	175	43.034^b^			

^a^.006 MS(Y3 Home Site) + .005 MS(Y6 Home Site) + .000 MS(Y6 Management Viva Site * Y3 Home Site) + .003 MS(Y6 Management Viva Site * Y6 Home Site) - .005 MS(Y3 Home Site * Y6 Home Site) - .002 MS(Y6 Management Viva Site * Y3 Home Site * Y6 Home Site) + .992 MS(Error)

^b^MS(Error)

^c^.745 MS(Y6 Management Viva Site * Y3 Home Site) + .974 MS(Y6 Management Viva Site * Y6 Home Site) - .864 MS(Y6 Management Viva Site * Y3 Home Site * Y6 Home Site) + .145 MS(Error)

^d^.583 MS(Y6 Management Viva Site * Y3 Home Site) + .492 MS(Y3 Home Site * Y6 Home Site) - .372 MS(Y6 Management Viva Site * Y3 Home Site * Y6 Home Site) + .297 MS(Error)

^e^.930 MS(Y6 Management Viva Site * Y6 Home Site) + .899 MS(Y3 Home Site * Y6 Home Site) - .884 MS(Y6 Management Viva Site * Y3 Home Site * Y6 Home Site) + .054 MS(Error)

^f^.820 MS(Y6 Management Viva Site * Y3 Home Site * Y6 Home Site) + .180 MS(Error)

^g^.893 MS(Y6 Management Viva Site * Y3 Home Site * Y6 Home Site) + .107 MS(Error)

^h^.972 MS(Y6 Management Viva Site * Y3 Home Site * Y6 Home Site) + .028 MS(Error)

**Table 5:** Factors impacting Y6 MCQ results: univariate analysis, tests of between-subjects effects

Source	Type III Sum of Squares	df	Mean Square	F	Sig.	Partial Eta Squared
Intercept	8.335	1	8.335	0.343	0.559	0.002
	5047.759	207.435	24.334^a^			
Y3 MCQ	562.901	1	562.901	22.942	0	0.1
	5079.02	207	24.536^b^			
Y3 OSCE	153.033	1	153.033	6.237	0.013	0.029
	5079.02	207	24.536^b^			
Y6 OSCE	213.847	1	213.847	8.716	0.004	0.04
	5079.02	207	24.536^b^			
Y6 Management Viva	179.999	1	179.999	7.336	0.007	0.034
	5079.02	207	24.536^b^			
Y6.MCQ.Site.rec	26.778	1	26.778	0.182	0.721	0.106
	226.879	1.544	146.942^c^			
Y3 Home Site	136.193	9	15.133	0.149	0.997	0.076
	1659.112	16.376	101.315^d^			
Y6 Home Site	67.343	3	22.448	0.148	0.921	0.217
	242.605	1.599	151.696^e^			
Y6.MCQ.Site.rec * Y3 Home Site	196.644	7	28.092	31.16	0	0.95
	10.243	11.362	.902^f^			
Y6.MCQ.Site.rec * Y6 Home Site	56.37	1	.	.	.	.
	.	.^g^	.			
Y3 Home Site * Y6 Home Site	471.76	14	.	.	.	.
	.	.^g^	.			
Y6.MCQ.Site.rec * Y3 Home Site * Y6 Home Site	0.271	1	0.271	0.011	0.916	0
	5079.02	207	24.536^b^			

^a^.008 MS(Y3 Home Site) + .013 MS(Y6 Home Site) - .001 MS(Y6.MCQ.Site.rec * Y3 Home Site) + .001 MS(Y6.MCQ.Site.rec * Y6 Home Site) - .007 MS(Y3 Home Site * Y6 Home Site) + .002 MS(Y6.MCQ.Site.rec * Y3 Home Site * Y6 Home Site) + .985 MS(Error)

^b^MS(Error)

^c^.965 MS(Y6.MCQ.Site.rec * Y3 Home Site) + 2.090 MS(Y6.MCQ.Site.rec * Y6 Home Site) - 2.161 MS(Y6.MCQ.Site.rec * Y3 Home Site * Y6 Home Site) + .106 MS(Error)

^d^2.245 MS(Y6.MCQ.Site.rec * Y3 Home Site) + .844 MS(Y3 Home Site * Y6 Home Site) - 2.516 MS(Y6.MCQ.Site.rec * Y3 Home Site * Y6 Home Site) + .428 MS(Error)

^e^2.126 MS(Y6.MCQ.Site.rec * Y6 Home Site) + .521 MS(Y3 Home Site * Y6 Home Site) - 2.254 MS(Y6.MCQ.Site.rec * Y3 Home Site * Y6 Home Site) + .607 MS(Error)

^f^.974 MS(Y6.MCQ.Site.rec * Y3 Home Site * Y6 Home Site) + .026 MS(Error)

^g^Cannot compute the error degrees of freedom using Satterthwaite’s method

The final set of analyses consisted of two linear regression models which aimed to identify whether the location of the respective examinations matching the students’ home site (that is, the student both learned in and was examined in the same location) had any meaningful impact on the students’ performance which would be reflected in exam results. This analysis also included for Y3 MCQ and OSCE as control variables.

The results demonstrate that the Y6OSCE results were positively impacted only byY3 OSCE, Y6Management Viva and Y6MCQ results, whereas Y3and Y6home site or whether Y6home site was different to Y6OSCE site did not have any statistically significant impact on grades (Table 6).

**Table 6:** Factors impacting Y6 OSCE results: linear regression

	B	Std. Error	Beta	t	Sig.	95%CI	
(Constant)	14.170	5.376		2.636	.009	3.580	24.759
Y3.MCQ[of]50	−.090	.145	−.032	−.626	.532	−.375	.194
Y3.Clinical[of]50	.584	.120	.262	4.879	.000	.348	.820
Y6 MCQ	.211	.060	.193	3.507	.001	.093	.330
Y6 Management Viva	.360	.044	.430	8.223	.000	.274	.446
Y6 Clinical Same Site	1.317	.850	.075	1.550	.122	−.357	2.991
Y6HomeSiteR/M	−.169	.644	−.012	−.262	.794	−1.438	1.101
Y3 HomeSiteR/M	1.728	.910	.091	1.898	.059	−.065	3.521

R2 = .558

Similar results were found regarding the factors impacting the Y6 Management Viva results. Only Y6OSCE and MCQ outcomes had positive and statistically significant impacts on Y6Management Viva grades, whereas Y3and Y6 home site or whether Y6 home site was different to Y6Management Viva site did not have any statistically significant impact on grades (Table 7).

**Table 7:** Factors impacting Y6 Management Viva results: linear regression

	B	Std. Error	Beta	t	Sig.	95%CI	
(Constant)	−2.485	7.113		−.349	.727	−16.497	11.526
Y3MCQ	.262	.187	.077	1.398	.163	−.107	.631
Y3 OSCE	.129	.163	.048	.793	.428	−.191	.450
Y6 MCQ	.243	.079	.185	3.074	.002	.087	.398
Y6 OSCE	.611	.074	.511	8.215	.000	.464	.758
Y6VivaSameSite	−1.262	1.163	−.059	− 1.085	.279	−3.554	1.030
Y6 Home Site R/M	1.592	.845	.093	1.885	.061	−.071	3.256
Y3 Home Site R/M	.315	1.207	.014	.261	.794	−2.062	2.692

R2 = .476

## Discussion

The current study aimed to identify how sites of students’ clinical learning and examinations (rural as compared with metropolitan) as well as other related factors may impact final year medical students’ results across three assessment types - MCQ, OSCE and Management Viva.

The results demonstrate that neither site of learning nor site of examination had any significant impact on the outcomes in any of these three assessments. These results are important from a number of perspectives, most relating to medical schools which operate rural clinical campuses. The main message is that students who study in rural clinical schools are neither advantaged nor disadvantaged compared to their counterparts studying in metropolitan clinical schools in terms of examination performance. The results also support the notion that, the selection and use of questions from a standardised Assessment Item Bank, combined with the use of identical grading criteria and Examiner Guidelines can minimise variation between examination sites. The literature regarding the impact of clinical setting on learning outcomes suggests that students may obtain better clinical experience due to exposure to more diverse cases in rural settings compared with metropolitan settings [11]. However, concerns regarding access to learning resources for students in rural clinical settings have also been raised, particularly in Australia [25].It is important to note that studying in rural campuses did not affect student performance in any of the assessment types undertaken in students’ final year of study. This is a critical finding as it alleviates concerns regarding limited access to learning resources in clinical settings [25]. A plausible explanation for this finding is the extensive and effective e-learning resources made available to students, which may mitigate the lack of local resources in rural clinical campuses [26–29].

From an assessment perspective, it is interesting to see that common biases impacting Management Viva and OSCE assessors, i.e. familiarity with the student, coming from similar background [19] have not been observed in the current study. A possible explanation is that examiners overall were not much biased, or biases might have been reduced or nullified by strategies discussed above. In addition, it is also possible that OBM2 [30], anew assessment moderation technique recently introduced to the OSCEs and Management Viva at UNSW, might have moderated any bias that might previously have existed. The OBM2 is a method that moderates examiner bias around borderline performance. That is, borderline marks awarded by lenient examiners are more likely to be converted to fail grades, whereas borderline marks awarded by stringent examiners tend to be converted to pass grades. Since this study used only the final OSCE and Management Viva (post OBM2) results, it was impossible to identify to what extent examiners’ bias was eliminated by the OBM2. Further studies are required to examine that issue.

Unlike previous similar studies, which did not control for the site of examination (for example: [31–33]), this study does consider in the analysis both the site of study and the site of examination, particularly trying to identify whether student-examiner familiarity has had any impact on examination outcomes [19]. The results demonstrate that either at the site level or at the setting level [rural / metropolitan, (Tables 6 and 7), students’ performance in assessments of clinical practice and knowledge (OSCE, MCQ and Management Viva) is independent of both the site of learning and the site of examination. These results suggest that the quality of teaching, learning and assessment is similar across all campuses of the UNSW Medicine program, which may be a finding that could be generalised to other similar medical programs. Further support for this conclusion is presented in the significant association across all main assessments (OSCE, MCQ and Management Viva) in both Year 3 and Year 6.

Despite the encouraging results, this study has a number of limitations, one being the sample size. This analysis included two categorical variables, each consisting of 12 categories. The sample size (*N* = 275) employed in this study could be considered too small for such data. However, increasing the size of the dataset by adding more cohorts would further reduce the statistical power of the study, because examinations and some examiners are different across cohorts. The remedy employed in this study was additional analyses, which collapsed sites into two categories (rural/metropolitan) as well as adding a binary variable indicating whether examination site was similar to the learning site (home site). The results of the additional analyses were in line with the underpowered analysis, which enhance our confidence that this study was not susceptible to either type 1 or type 2 statistical errors.

It is noted that extracting information about the reliability or any other psychometric properties of the assessments was outside the scope of this study. The reason for that is that the purpose of this study was to serve overall scores, not psychometric characteristics of the various measures included.

It is also acknowledged that there are a number of factors that could have resulted in differences across rural/metropolitan sites that were outside the scope of the current investigation. For example, possible mechanisms or influential factors relating to the association between the locations (rural vs. Metropolitan) and assessment outcomes may be related to difference in examiners’ characteristics across sites for example seniority (senior examiners award lower marks than junior examiners), experience in assessing (the more experienced the examiners the lower the marks awarded) [34]. Furthermore, the difference in assessment outcomes may also be related to differences in technical practices applied by different clinicians, which may relate to professional experience [35]. Nonetheless, exploring the mechanisms underlying the potential biases in assessment outcomes across examination sites was not within the scope of the current study. Further research is required to address these issues.

## Conclusions

This study demonstrated that studying in rural clinical schools neither advantages nor disadvantages medical students learning outcomes across a range of the main assessment types in the UNSW Medicine program (MCQ, OSCE and Management Viva). It is suggested that some of the supposed disadvantages inherent in rural campuses, are effectively mitigated by perceived advantages in regard to more intensive interaction with patients and the general and medical communities in those sites, as well as effective e-learning resources and moderation of assessment grades. The results of this study also support that the selection and use of questions from a standardised Assessment Item Bank, combined with the use of identical grading criteria and Examiner Guidelines can minimise variation between examination sites. Further studies may examine in more detail the specific factors that enable the success of rural medical training.

## Data Availability

Data may be available by request submitted to the corresponding author, subject to the approval the Human Research Ethics Committee of the University of New South Wales.

## Abbreviations

MCQ: Multiple Choice Questions
OSCE: Objective Structured Clinical Examination
UNSW: University of New South Wales
